# Differential Roles of Tumor Necrosis Factor Ligand Superfamily Members as Biomarkers in Pancreatic Cancer

**DOI:** 10.3390/jcm7070175

**Published:** 2018-07-13

**Authors:** Ines Pombeiro, Sven H. Loosen, Sanchari Roy, Florian Schueller, Lukas Niewenhuisen, Mark Luedde, Mihael Vucur, Frank Tacke, Marcel Binnebösel, Wenzel Schoening, Christian Trautwein, Tom Luedde, Ulf Peter Neumann, Christoph Roderburg

**Affiliations:** 1 Department of Medicine III, University Hospital RWTH Aachen, Pauwelsstrasse 30, 52074 Aachen, Germany; ipombeiro@ukaachen.de (I.P.); sloosen@ukaachen.de (S.H.L.); sroy@ukaachen.de (S.R.); fschueller@ukaachen.de (F.S.); lniewenhuisen@ukaachen.de (L.N.); frtacke@ukaachen.de (F.T.); ctautwein@ukaachen.de (C.T.); croderburg@ukaachen.de (C.R.); 2 Division of Gastroenterology, Hepatology and Hepatobiliary Oncology, University Hospital RWTH Aachen, Pauwelsstrasse 30, 52074 Aachen, Germany; mvucur@ukaachen.de; 3 Department of Internal Medicine III, University of Kiel, Schittenhelmstrasse 12, 24105 Kiel, Germany; Mark.Luedde@uksh.de; 4 Department of Surgery, University Hospital RWTH Aachen, Pauwelsstrasse 30, 52074 Aachen, Germany; mbinnebosel@ukaachen.de (M.B.); wschoening@ukaachen.de (W.S.); upneumann@ukaachen.de (U.P.N.)

**Keywords:** TWEAK, APRIL, TNF, serum, pancreatic cancer, biomarker, prognosis

## Abstract

The tumor necrosis factor–related weak inducer of apoptosis (TWEAK) belongs to the tumor necrosis factor ligand superfamily, which was shown to play an important role in inflammatory and malignant gastrointestinal diseases, including colitis or colorectal cancer. However, in contrast to other members of the TNF ligand superfamily, its role as a biomarker in pancreatic cancer is currently unknown. We analyzed serum levels of A proliferation-inducing ligand (APRIL) and TWEAK in 134 patients with pancreatic cancer. Results were compared with 50 healthy controls and correlated with clinical data. Intratumoral expression of APRIL and TWEAK in pancreatic cancer was analysed using the datasets made available by the TCGA-LIHC project. APRIL serum levels were significantly elevated in patients with pancreatic cancer compared to healthy controls, which is in line with previous findings. Notably, the diagnostic accuracy of circulating APRIL levels was similar to CA19-9, an established tumor marker for pancreatic cancer. In contrast, serum concentrations of TWEAK were decreased in pancreatic cancer patients. Interestingly, no differences in TWEAK concentrations became apparent between different clinical subgroups of pancreatic cancer. Moreover, within our cohort of patients, TWEAK levels did not correlate with the patients’ prognosis and the diagnostic as well as prognostic potential of TWEAK was lower than CA 19-9, when analyzed in this setting. Finally, using data from the TCGA-LIHC project, we demonstrate that expression levels of TWEAK and APRIL represent prognostic markers for patients’ survival according to Kaplan-Meier curve analyses. TWEAK and APRIL serum concentrations are regulated differently in patients with pancreatic cancer, highlighting diverse roles of variant TNF ligands in this type of cancer.

## 1. Introduction

Pancreatic ductal adenocarcinoma (PDAC) is the 12th most common cancer worldwide [1]. Due to its unfavourable prognosis and the lack of effective treatment options at later stage of disease, early diagnosis is essential to optimize possible treatment options and to improve patients’ outcome [2]. Therefore, new non-invasive diagnostic biomarkers could be a valuable addition to the existing diagnostic work-up algorithms. Moreover, prognostic biomarkers could represent a useful tool to divide pancreatic cancer patients into different subgroups, providing each patient an optimal personalized therapeutic approach according to the individual likelihood to benefit from a specific surgical, chemotherapeutic, or conservative treatment [3]. Currently, carbohydrate antigen (CA) 19-9 is the only biomarker for PDAC that is approved by the US Food and Drug Administration (FDA), but its diagnostic sensitivity and specificity is poor [4,5,6].

Various reports have suggested a function of tumour necrosis factor (TNF) in the tumorigenesis of PDAC [7,8]. The TNF superfamily ligands represent a class of type II transmembrane proteins, exerting their biological activity as non-covalently bound trimers [9]. Activation of TNF receptor (TNFR) members has been shown to play a pivotal role during infectious and inflammatory diseases. Besides TNF, different members of the TNF ligand superfamily including A proliferation inducing ligand (APRIL) and tumor necrosis factor–related weak inducer of apoptosis (TWEAK) have been implicated in the pathogenesis of cancer [10,11,12,13].

In this context, different authors suggested that TWEAK promotes of apoptosis, cell growth as well as angiogenesis. Blocking TWEAK in pancreatic cancer cell lines resulted in a 22–65% cell growth inhibition of these cells, highlighting the therapeutic potential of this specific TNFR ligand in pancreatic cancer [14]. In line, out of six patients, treated with Enavatuzumab, a humanized IgG1 antibody to the TWEAK receptor, four demonstrated an objective tumor response. Moreover, some of these ligands have been suggested to play a role as biomarkers in malignant disease. As such, elevated serum levels of APRIL have been described in patients with pancreatic cancer [15,16,17] and were suggested to be a diagnostic marker. Moreover APRIL levels were proposed to serve as a potential prognostic biomarker to assess the outcome of these patients [18]. Despite the fact that alterations of TWEAK levels were found in patients with different cancer as well as in patients with inflammatory or cardiovascular diseases [19,20,21], the role of TWEAK serum concentrations in patients with PDAC remains unknown.

To analyse serum concentrations of TWEAK in patients with PDAC and to evaluate a potential diagnostic or prognostic impact of TWEAK serum levels in these patients, we measured its serum concentrations in 134 patients with pancreatic cancer at different stages of disease. Moreover, TWEAK serum concentrations were correlated to patients’ characteristics such as tumour stage, survival and routinely accessed laboratory parameters.

## 2. Patients and Methods

### 2.1. Study Design and Patient Characteristics

This observational cohort study was designed to evaluate TWEAK as a diagnostic or prognostic serum marker in patients with pancreatic cancer. Patients were enrolled from University Hospital RWTH Aachen and were prospectively recruited. 134 patients with pancreatic cancer (APRIL cohort (*n* = 31): 58.1% male, 41.9% female, median age: 59 years, range 26–83 years; TWEAK cohort (*n* = 134): 56.7% male, 43.3% female, median age: 67.5 years, range 26–84 years; see Table 1 and Table 2) were diagnosed based on patients’ history, physical examination (silent jaundice, weight loss), imaging techniques (CT, MRI) as well as laboratory tests (elevated AST, ALT, AP, GGT, bilirubin, and CA 19-9 concentration) and were further confirmed histopathologically after tumor resection. As a control population, we analyzed 50 healthy, cancer-free blood donors with normal values for blood count, C-reactive protein, and liver enzymes. The study protocol was approved by the local ethics committee and conducted in accordance with the ethical standards laid down in the Declaration of Helsinki (ethics committee of the University Hospital Aachen, RWTH University, Aachen, Germany). Written informed consent was obtained from the patients.

### 2.2. Determination of Serum APRIL and TWEAK Levels

Circulating levels of APRIL were determined using a commercial enzyme-linked immunosorbent assay (ELISA) according to the manufacturers’ instructions (Product No. SEB750Hu, USCN Life Science, Wuhan, China). The APRIL-ELISA represents a sandwich enzyme immunoassay for the quantitative measurement of APRIL in human serum, plasma, and other biological fluids. TWEAK serum concentrations were likewise analyzed using a commercially available ELISA following the manufacturers’ instructions (Product No. O43508, Ray Biotech, Norcross, GA, USA). The TWEAK-ELISA is a standard sandwich enzyme immunoassay for the quantitative measurement of TWEAK in human serum, plasma, and cell culture supernatants.

### 2.3. Statistical Analysis

Statistical analyses have been performed as recently described in detail [22,23,24]. In summary, data are given as median and range to reflect the skewed distribution of analysis on human samples. The Mann-Whitney-U-test and, for multiple comparisons, the Kruskal-Wallis-H-Test were used. Box plot graphics display a statistical summary of the median, quartiles, and ranges. Correlations analyses were performed using the Spearman correlation tests. Kaplan-Meier curves were plotted to display the impact on the overall survival (OS). The optimal cut-off value for the identification of patients with an impaired OS was established using a recently published biometric software, which fits Cox proportional hazard models to the dichotomized survival status (dead vs. alive) and the survival variable (survival time). The optimal cut-off is hereby defined as the point with the most significant (log-rank test) split [25]. The prognostic relevance of serum TWEAK was further tested using univariate Cox-regression analysis. ROC curves were generated by plotting sensitivity against 1-specificity. All statistical analyses were performed with SPSS (SPSS 23, Chicago, IL, USA).

### 2.4. TGCA-PAAD

The raw data (count data) for messenger RNA data set were downloaded from (https://portal.gdc.cancer.gov/projects/TCGA-PAAD). The miRNA datasets consisted of 178 samples that were annotated regarding “tumor stage” (21 stage I, 146 stage II, 3 stage III, 5 stage IV; for 3 samples, tumor stage was not reported. Differentially expressed genes were identified by using linear models and moderated F- and t-statistics. Clinical data including the survival time of the patients were also retrieved from TCGA data portal cBioPortal (http://www.cbioportal.org/index.do). Survival cut off value was analyzed using the tool Cutoff Finder (http://molpath.charite.de/cutoff/) and used the cut-off value to separate the patients’ survival on “low” and “high” expression. The percent survival was calculated by GraphPad Prism Software.

## 3. Results

### 3.1. APRIL Serum Concentrations Are Elevated in Patients with Pancreatic Cancer

**Figure 1:**
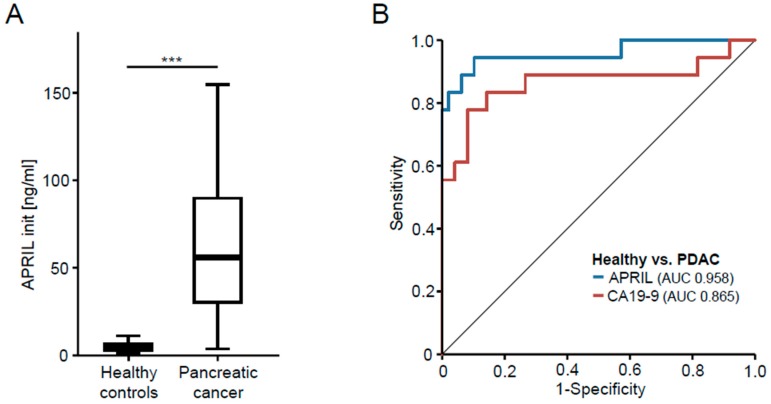
APRIL serum concentrations are elevated in patients with pancreatic cancer. (**A**) Serum concentrations of TWEAK were analyzed by enzyme-linked immunosorbent assay (ELISA) in patients with pancreatic cancer and healthy blood donors as controls; (**B**) ROC curve analysis comparing the diagnostic value of APRIL and CA19-9 for pancreatic cancer.

Elevated serum levels of APRIL were recently described in patients with PDAC [15,16,17]. To validate the suitability of our cohort of patients with pancreatic cancer as well as our general analysis set-up to determine differences in serum concentrations of TNF ligands, we first measured APRIL serum concentrations in a subgroup of patients and healthy controls (patients’ characteristics are given in Table 1). Notably, this analysis revealed significantly higher levels of APRIL in PDAC patients compared to healthy controls (Figure 1A). Based on this strong regulation, we next attempted to compare the diagnostic accuracy of APRIL and CA19-9 for pancreatic cancer. In this analysis, APRIL displayed an AUC value of 0.958 compared to 0.865 for CA19-9 (Figure 1B). Thus, these analyses, which are in line with existing data, prove the suitability of our system to detect a potential regulation in serum concentrations of members of the TNF ligand superfamily in patients with pancreatic cancer.

### 3.2. TWEAK Serum Concentrations Are Decreased in Patients with Pancreatic Cancer but Independent of the Disease Stage

**Figure 2:**
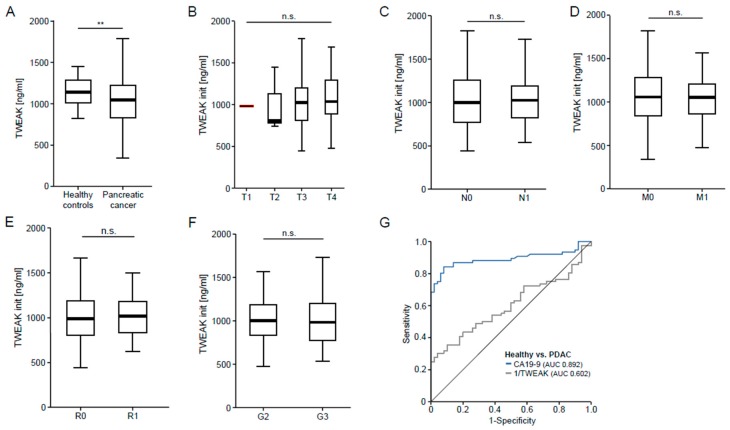
TWEAK serum concentrations are decreased in patients with pancreatic cancer but are independent of the disease stage. (**A**) Serum TWEAK concentrations were measured in patients with pancreatic cancer and healthy blood donors as controls; (**B**–**F**) TWEAK levels were unaltered in patients with different T-status, nodal positive vs. negative disease, metastasized vs. non-metastasized disease, R0 and R1 resected patients, and different tumor gradings; (**E**) TWEAK levels were unaltered in patients with R0 vs. R1 resection; (**G**) ROC curve analysis comparing the diagnostic value of TWEAK and CA19-9 for pancreatic cancer.

Next, we aimed to identify a potential role of TWEAK serum levels as a novel biomarker for pancreatic cancer. We therefore analyzed serum levels of TWEAK in our cohort of 134 PDAC patients and compared them to healthy controls (patients’ characteristics are given in Table 2). Unexpectedly, this analysis revealed significantly lower serum levels of TWEAK in PDAC patients compared to healthy controls (Figure 2A). Subsequently, we compared TWEAK serum concentrations between different disease stages (T stages, nodal negative vs. positive disease, non-metastasized vs. metastasized disease, moderately vs. poorly differentiated tumors). However, this analysis revealed no significant differences between these subgroups of patients (Figure 2B–F). Moreover, serum levels of TWEAK were unaltered in patients with incomplete tumor resection (R1) compared to patients with complete tumor resection (R0) (Figure 2E) and did not correlate with clinical symptoms of pancreatic cancer such as fatigue, pain or impaired ECOG performance status (Supplementary Figure S1). To unravel potential mechanisms involved in the regulation of serum TWEAK concentrations, we next analyzed potential correlations between TWEAK serum levels and routinely used laboratory markers. In this analysis, TWEAK serum concentrations negatively correlated with serum levels of C-reactive protein (*r* = −0.242, *p* = 0.010), which is contradictory to the assumption that tumors develop an inflammatory microenvironment. It must be noted however that in our cohort the TWEAK serum levels were lower in patients suffering from PDAC than in healthy controls. Moreover, TWEAK levels correlated negatively with serum levels of bilirubin (*r* = −0.179, *p* = 0.04), GGT (*r* = −0.212, *p* = 0.022), and AP (*r* = −0.298, *p* = 0.001), which are commonly understood as markers for cholestasis in pancreatic cancer (Table 3). Finally, we compared the diagnostic accuracy of TWEAK and CA19-9 for pancreatic cancer. In this analysis, TWEAK displayed an AUC value of only 0.602 compared to 0.892 for CA19-9 (Figure 2G). In summary, these results suggest that, in contrast to APRIL, TWEAK serum levels are not relevantly regulated in patients with PDAC and are therefore unsuitable as a diagnostic marker for this setting.

### 3.3. TWEAK Serum Concentrations Do Not Predict Overall Survival in Patients with Pancreatic Cancer

**Figure 3:**
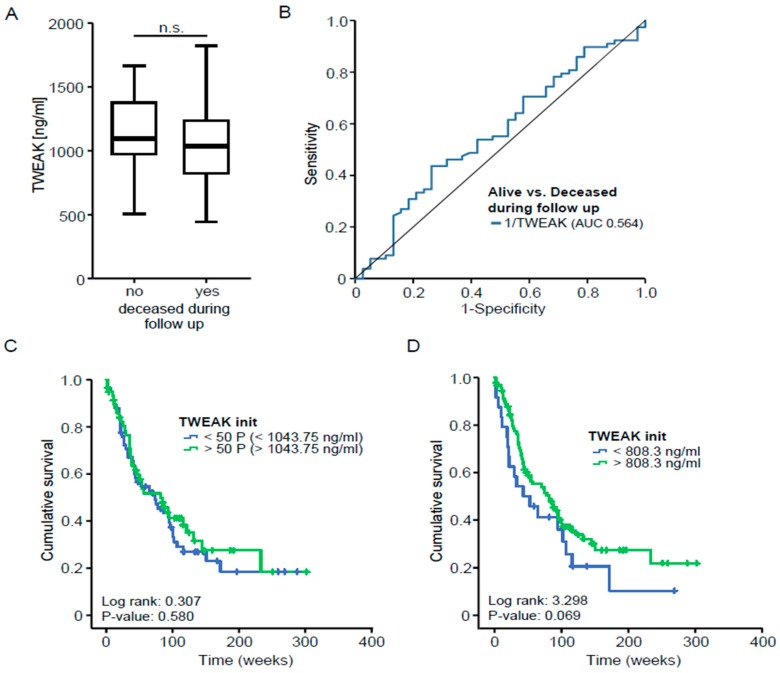
TWEAK serum concentrations do not predict survival in patients with pancreatic cancer. (**A**) Serum TWEAK concentrations were measured in patients that succumbed to death and survivors; (**B**) ROC curve analyses determining the prognostic value of TWEAK in patients with pancreatic cancer; (**C**) Kaplan-Meier curve analysis with respect to patients’ serum TWEAK concentrations (cut-off determined by using the median); (**D**) Kaplan-Meier curve analysis with respect to patients’ serum TWEAK concentrations (ideal cut-off value).

Several studies have recently demonstrated a role of TNF ligand superfamily members as prognostic biomarkers in various benign and malignant diseases. As an example, it was shown that elevated serum APRIL levels are indicative for tumor recurrence and an impaired prognosis after resection of PDAC. To identify a potential association between TWEAK serum levels and patients’ outcome, we compared TWEAK serum concentrations in patients that succumbed to death during the follow-up period and survivors. Notably, this analysis revealed similar TWEAK serum levels between these subgroups (Figure 3A), which was confirmed by an AUC of 0.564 for TWEAK when used to distinguish between survivors and non-survivors (Figure 3B). To analyze the prognostic accuracy of TWEAK serum concentrations, we next compared the overall survival of patients with high or low initial TWEAK levels (above or below the 50th percentile). However, Kaplan-Meier curve analysis revealed no significant difference between these groups (Figure 3C). We next established an ideal prognostic TWEAK cut-off value by fitting Cox proportional hazard models to the survival status and the survival time and tested for the most significant log-rank test as recently described [25]. This analysis revealed that a TWEAK serum level of 808.3 ng/mL best distinguishes between patients with a good or poor postoperative prognosis. However, despite a strong trend towards an impaired prognosis in patients with low TWEAK serum levels (below 808.3 ng/mL), this ideal cut-off value was still unable to significantly identify a subgroup of patients with an impaired overall survival (Figure 3D). In line, univariate Cox-regression analysis revealed that initial TWEAK serum concentrations were unable to predict patients’ outcome after tumor resection (Hazard ratio: 1.000, *p* = 0.807), suggesting that TWEAK serum levels do not reflect the postoperative prognosis of PDAC patients undergoing surgical tumor resection.

### 3.4. Tissue APRIL and TWEAK Expression as a Predictor for Patients Survival

**Figure 4:**
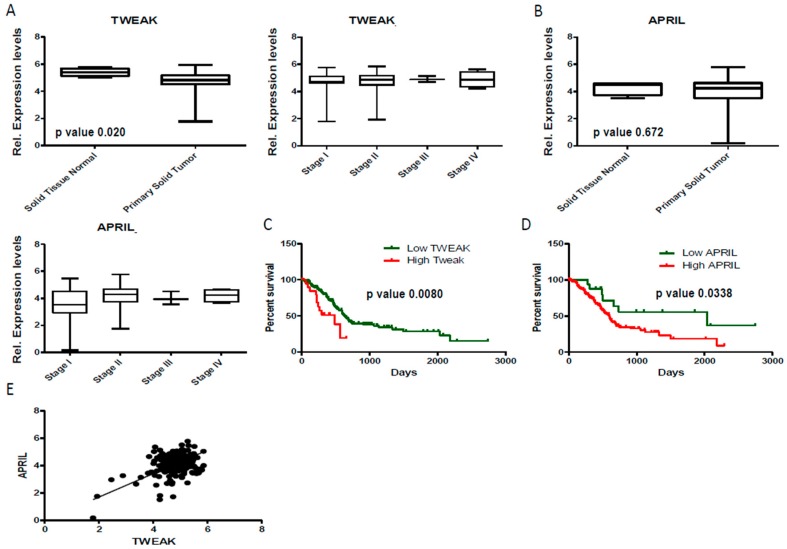
Low intratumoral TWEAK expression levels predict an unfavorable survival in patients with pancreatic cancer. The raw data (count data) for messenger RNA data set were downloaded from (https://portal.gdc.cancer.gov/projects/TCGA-PAAD). (**A**) Relative TWEAK expression levels are depicted; (**B**) Relative APRIL expression levels are depicted (**C**) Kaplan-Meier curve analysis with respect to patients’ TWEAK expression; (**D**) Kaplan-Meier curve analysis with respect to patients’ TWEAK expression; (**E**) Correlation analysis between relative TWEAK and APRIL expression.

Based on these results we next attempted at examining a molecular function of APRIL and TWEAK in pancreatic cancer as well as the role of intratumoral TWEAK and APRIL expression as a predictor for patients survival. Considering the lack of corresponding tissue samples for the analyzed serum samples, we analyzed the expression of APRIL and TWEAK within the datasets made available by the TCGA-LIHC project. We therefore downloaded the raw data (count data) the messenger RNA data set. The datasets consisted of 178 samples that were annotated regarding “tumor stage” (4 normal, 21 stage I, 146 stage II, 3 stage III, 5 stage IV; for 3 samples tumor stage was not reported). These results showed that TWEAK but not APRIL was significantly lower expressed in tumor vs non-tumor tissue (*p* = 0.020 and 0.60; respectively; Figure 4A,B), being fully consistent with the results we presented regarding lower levels of TWEAK in serum of patients with pancreatic cancer (Figure 2A). Strikingly low serum concentrations of TWEAK but not APRIL turned out as a significant prognostic marker for patients’ survival according to Kaplan-Meier curve analysis (Figure 4C,D), highlighting the relevance of TWEAK in the pathogenesis of pancreatic cancer. Finally, intratumoral TWEAK and APRIL expression were strongly correlated, underlining that common mechanism might be involved in the regulation of these TNFR ligands in pancreatic cancer.

## 4. Discussion

TNF has been widely implicated in the pathophysiology of different cancers including pancreatic carcinoma [7,8]. Increased levels of APRIL, a bona fide member of the TNF ligand superfamily, were previously demonstrated in the serum of patients with pancreatic cancer and were shown to correlate with early tumor recurrence and an impaired patients’ prognosis [15,16,17], suggesting that other members of the TNF superfamily might hold a similar role. In this context, elevated tissue expression levels of TWEAK were reported from patients with pancreatic cancer when compared to patients with chronic pancreatitis or healthy controls. In this study, we analyzed TWEAK serum levels in a large and well characterized cohort of patients with pancreatic cancer at different disease stages. Unexpectedly, in our cohort of patients, TWEAK levels were independent of the disease stage and did not reflect patients’ outcome, highlighting that different members of the TNF ligand superfamily (such as APRIL or TWEAK) might have different roles in the pathophysiology of this disease and might reflect different aspects of the pathophysiology of pancreatic cancer when used as a biomarker in this setting.

PDAC represents one of the most devastating diagnoses to date. Only in case of early diagnosis are curative treatment approaches available [2]. Thus, diagnostic modalities allowing tumour detection at an early time-point might have an important role in the treatment of PDAC. However, at present, besides measurement of CA19-9, no serum-based (and therefore easily accessible) biomarker has a sufficient sensitivity or specificity to be used in clinical routine [26]. Recent research in the context of PDAC and other cancers suggest that innovative molecules such as TNF receptor ligands might overcome these limitations [26]. In this study, we demonstrate that the diagnostic accuracy of APRIL is superior to that of CA19-9 measurements in patients with PDAC. Besides being used in the context of diagnosis, treatment predictive and prognostic biomarkers might have an essential role in providing an optimal and personalized treatment to patients with pancreatic cancer. Despite a major scientific effort during the last decades, the ideal biomarker for these purposes has not been identified yet. The combination of different markers as diagnostic or prognostic indices appears promising. One might therefore speculate that a combination of different TNF ligand superfamily members with CA 19-9 might further improve the diagnostic and prognostic accuracy of these markers. Nevertheless, larger studies, featuring a longitudinal and multicentre design are needed to validate these novel biomarkers and to further evaluate the clinical potential of such marker combinations.

Based on their deep integration into the pathophysiology of many diseases, the use of TNF ligand superfamily members as biomarkers represents a biologically plausible concept. To prove the suitability of our cohort of patients for detecting differences in TNF ligand superfamily members in PDAC-patients and healthy controls, we first analyzed concentrations of APRIL, which is known to be deregulated in pancreatic cancer in a smaller subgroup of our cohort. Importantly, these analyses were in line to all previous reports, enabling us to analyze serum levels of TWEAK in all patients included in this study. Conversely to APRIL, TWEAK levels were significantly lower in patients with pancreatic cancer when compared with healthy controls. Moreover, while levels of APRIL have been demonstrated to reflect disease characteristics, no similar correlation was detected for TWEAK in this context. These striking differences between different members of the TNF ligand superfamily are confirmed by similar differences between these and other members of the superfamily in patients with critical illness and sepsis. As such, we recently demonstrated that both TNF and APRIL serum levels are up-regulated in patients with sepsis, while serum levels of TWEAK were down-regulated and serum levels of GITRL were unchanged [21,27]. Thus, it seems likely that the observation on a distinct regulation of APRIL and TWEAK is not a “random phenomenon” but rather reflects a different role of these molecules in the pathophysiology of pancreatic cancer. In this context, the role of TWEAK in the pathophysiology of pancreatic cancer is of especial interest, as it has been demonstrated that pronounced therapeutic effects are achievable with soluble TWEAK-antibodies in a variety of disease models [28]. Consequently, TWEAK-specific antibodies are currently being tested in the context of inflammatory diseases and in patients with solid tumors [28]. Besides anti-TWEAK antibodies, Tigatuzumab, a humanized version of the agonistic murine monoclonal antibody TRA-8, directed against TRAIL, another member of the TNF ligand superfamily, is under investigation in patients with pancreatic cancer [29], highlighting the therapeutic potential of these ligands.

Similar to sepsis, PDAC results in local and systemic inflammation [30]. Thus, our data on a different regulation of different members of the TNF ligand superfamily might reflect different roles of these proteins in the complex regulation of the inflammatory response during the disease progress of patients suffering from PDAC. In this context, it is important to note that PDAC results in a strong activation of the NF-KB pathway, which is the common signaling pathway of all members of the TNF ligand superfamily. NF-κB also functions as a key link between pancreatic inflammation and cancer [31]. It was demonstrated that macrophages from patients with chronic pancreatitis secrete increased amounts of TNF [32]. Thus, the differential regulation of different TNF ligand superfamily members might reflect an adaption mechanism of activated macrophages towards different inflammatory stimuli during the development of PDAC.

In summary, our data clearly argue against a potential use of TWEAK serum levels as a biomarker in patients with PDAC. Nevertheless, from a basic scientific view, our data suggest that different TNF superfamily members might exert different roles in the development of PDAC. These data should trigger further research using e.g., animal models to shed light on the specific roles of TWEAK and other TNFR ligands in in PDAC.

## Figures and Tables

**Table 1:** Study population in the A proliferation inducing ligand (APRIL) cohort.

Parameter	
Number	31
Sex (male/female)	18/13 (58.1/41.9%)
Age median (range) (years)	59 (26–83)
Staging	
T1–T2–T3–T4	0–0–14–3 (0–0–45.2–9.7%)
N0–N1	5–11 (16.1–35.5%)
M0–M1	16–12 (51.6–38.7%)
G2–G3	11–7 (35.5–22.6%)
R0–R1–R2	9–3–1 (29.0–9.7–3.2%)
UICC	
I–II–III–IV	0–17–1–12 (0–54.8–3.2–38.7%)
ECOG	
ECOG 0	11 (35.5%)
ECOG I	17 (54.8%)
ECOG II	3 (9.7%)
ECOG III	0 (0%)
ECOG IV	0 (0%)
Fatigue	
None	10 (32.2%)
Low	1 (3.2%)
Medium	9 (29.0%)
High	11 (35.5%)
Pain scale	
0	13 (41.9%)
1–4	7 (22.6%)
5–7	9 (29.0%)
8–10	2 (6.5%)
CRP median (range) (mg/L)	8.0 (0.00–134.00)
Bilirubin median (range) (mg/L)	0.60 (0.20–26.20)
GGT median (range) (U/L)	218 (21–2138)
AP median (range) (U/L)	167 (64–1574)
AST median (range) (U/L)	28 (17–202)
ALT median (range) (U/L)	35 (15–451)
CEA median (range) (µg/L)	3.5 (1.2–16.0)
CA 19-9 median (range) (kU/L)	46 (2–10,000)
Creatinine median (range) (mg/dL)	0.8 (0.5–3.3)
WBC median (range) (cells/µg)	7.4 (5.4–10.5)

**Table 2:** Study population in the tumor necrosis factor–related weak inducer of apoptosis (TWEAK) cohort.

Parameter	
Number	134
Sex (male/female)	76/58 (56.7/43.3%)
Age median (range) (years)	67.50 (26–84)
Tumor type	
IPMN	8 (6%)
PanIN	10 (7.5%)
PDAC	116 (86.6%)
Staging	
T1–T2–T3–T4	2–3–66–8 (1.5–2.2–49.3–6%)
N0–N1	23–52 (17.2–38.9%)
M0–M1	88–36 (65.7–26.9%)
G2–G3	43–33 (32.1–24.6%)
R0–R1–R2	42–19–3 (31.3–14.2–2.2%)
UICC	
I–II–III–IV	4–64–4–36 (3–47.8–3–26.9%)
ECOG	
ECOG 0	60 (44.8%)
ECOG I	44 (32.8%)
ECOG II	39 (29.1%)
ECOG III	6 (4.5%)
ECOG IV	1 (0.7%)
Fatigue	
None	45 (33.6%)
Low	19 (14.1%)
Medium	17 (12.7%)
High	21 (15.7%)
Pain scale	
0	64 (47.8%)
1–4	12 (9%)
5–7	20 (14.9%)
8–10	6 (4.5%)
CRP median (range) (mg/L)	7.75 (0–237)
Bilirubin median (range) (mg/dL)	0.57 (0.15–26.20)
GGT median (range) (U/L)	107 (10–2138)
AP median (range) (U/L)	125.5 (39–1574)
AST median (range) (U/L)	27 (13–418)
ALT median (range) (U/L)	35 (7–569)
CEA median (range) (µg/L)	3 (0.22–76.30)
CA 19-9 median (range) (kU/L)	86.15 (0.6–266,567)
Creatinine median (range) (mg/dL)	0.82 (0.4–53.0)
WBC median (range) (cells/µL)	7.4 (2.7–23.30)

CRP, C-reactive protein; WBC, white blood cell count; AST, aspartate transaminase; ALT alanine transaminase; GGT, γ-glutamyl-transpeptidase; AP, alkaline phosphatase; CEA, carcinoembryonic antigen; CA 19-9 carbohydrate antigen.

**Table 3:** Correlations of TWEAK and variant laboratory markers.

Parameter	*r*	*p*
AST	−0.084	0.336
ALT	−0.188	0.89
WBC	0.062	0.477
Bilirubin	−0.179	0.04
GGT	−0.212	0.022
AP	−0.298	0.001
Albumin	0.338	0.008
CRP	−0.242	0.010
Creatinine	0.085	0.333
CEA	−0.050	0.664
CA 19-9	−0.072	0.507

*r*, correlation coefficient; *p*, *p*-value; *r* and *p*-values by Spearman rank correlation.

## Supplementary Materials

The following are available online at http://www.mdpi.com/2077-0383/7/7/175/s1. Figure S1: Serum levels of TWEAK in different subgroups of patients with pancreatic cancer.

## References

[1] Kamisawa T., Wood L.D., Itoi T., Takaori K. (2016). Pancreatic cancer. Lancet.

[2] Schrag D. (2016). Optimizing Treatment for Locally Advanced Pancreas Cancer: Progress but No Precision. JAMA.

[3] Chiaravalli M., Reni M., O’Reilly E.M. (2017). Pancreatic ductal adenocarcinoma: State-of-the-art 2017 and new therapeutic strategies. Cancer Treat. Rev..

[4] Goonetilleke K.S., Siriwardena A.K. (2007). Systematic review of carbohydrate antigen (CA 19-9) as a biochemical marker in the diagnosis of pancreatic cancer. Eur. J. Surg. Oncol..

[5] Zhang Y., Yang J., Li H., Wu Y., Zhang H., Chen W. (2015). Tumor markers CA19-9, CA242 and CEA in the diagnosis of pancreatic cancer: A meta-analysis. Int. J. Clin. Exp. Med..

[6] Loosen S.H., Neumann U.P., Trautwein C., Roderburg C., Luedde T. (2017). Current and future biomarkers for pancreatic adenocarcinoma. Tumour Biol..

[7] Carbone C., Melisi D. (2012). NF-κB as a target for pancreatic cancer therapy. Expert Opin. Ther. Targets.

[8] Friess H., Guo X.Z., Nan B.C., Kleeff J., Buchler M.W. (1999). Growth factors and cytokines in pancreatic carcinogenesis. Ann. N. Y. Acad. Sci..

[9] Peschon J.J., Slack J.L., Reddy P., Stocking K.L., Sunnarborg S.W., Lee D.C., Russell W.E., Castner B.J., Johnson R.S., Fitzner J.N. (1998). An essential role for ectodomain shedding in mammalian development. Science.

[10] Winkles J.A., Tran N.L., Brown S.A., Stains N., Cunliffe H.E., Berens M.E. (2007). Role of TWEAK and Fn14 in tumor biology. Front. Biosci..

[11] Winkles J.A., Tran N.L., Berens M.E. (2006). TWEAK and Fn14: New molecular targets for cancer therapy?. Cancer Lett..

[12] Haiat S., Billard C., Quiney C., Ajchenbaum-Cymbalista F., Kolb J.P. (2006). Role of BAFF and APRIL in human B-cell chronic lymphocytic leukaemia. Immunology.

[13] Ware C.F. (2000). APRIL and BAFF connect autoimmunity and cancer. J. Exp. Med..

[14] Yoriki R., Akashi S., Sho M., Nomi T., Yamato I., Hotta K., Takayama T., Matsumoto S., Wakatsuki K., Migita K. (2011). Therapeutic potential of the TWEAK/Fn14 pathway in intractable gastrointestinal cancer. Exp Ther Med..

[15] Wang F., Chen L., Mao Z.B., Shao J.G., Tan C., Huang W.D. (2008). Lentivirus-mediated short hairpin RNA targeting the APRIL gene suppresses the growth of pancreatic cancer cells in vitro and in vivo. Oncol. Rep..

[16] Wang F., Chen L., Ding W., Wang G., Wu Y., Wang J., Luo L., Cong H., Wang Y., Ju S. (2011). Serum APRIL, a potential tumor marker in pancreatic cancer. Clin. Chem. Lab. Med..

[17] Han L., Zhang W., Song F., Guo Y., Guo K., Zhou W. (2014). Soluble aproliferationinducing ligand (sAPRIL), a novel serum biomarker predicting the recurrence and metastasis of pancreatic adenocarcinoma after surgery. Mol. Med. Rep..

[18] Lam E.T., Eckhardt S.G., Messersmith W., Jimeno A., O’Bryant C.L., Ramanathan R.K., Weiss G.J., Chadha M., Oey A., Ding H.T. (2018). Phase I Study of Enavatuzumab, a First-in-Class Humanized Monoclonal Antibody Targeting the TWEAK Receptor, in Patients with Advanced Solid Tumors. Mol. Cancer Ther..

[19] Chorianopoulos E., Rosenberg M., Zugck C., Wolf J., Katus H.A., Frey N. (2009). Decreased soluble TWEAK levels predict an adverse prognosis in patients with chronic stable heart failure. Eur. J. Heart Fail..

[20] Park M.C., Chung S.J., Park Y.B., Lee S.K. (2008). Relationship of serum TWEAK level to cytokine level, disease activity, and response to anti-TNF treatment in patients with rheumatoid arthritis. Scand. J. Rheumatol..

[21] Roderburg C., Benz F., Schuller F., Pombeiro I., Hippe H.J., Frey N., Trautwein C., Luedde T., Koch A., Tacke F. (2016). Serum Levels of TNF Receptor Ligands are Dysregulated in Sepsis and Predict Mortality in Critically Ill Patients. PLoS ONE.

[22] Loosen S.H., Roderburg C., Kauertz K.L., Pombeiro I., Leyh C., Benz F., Vucur M., Longerich T., Koch A., Braunschweig T. (2017). Elevated levels of circulating osteopontin are associated with a poor survival after resection of cholangiocarcinoma. J. Hepatol..

[23] Loosen S.H., Benz F., Niedeggen J., Schmeding M., Schuller F., Koch A., Vucur M., Tacke F., Trautwein C., Roderburg C. (2016). Serum levels of S100A6 are unaltered in patients with resectable cholangiocarcinoma. Clin. Transl. Med..

[24] Roderburg C., Urban G.W., Bettermann K., Vucur M., Zimmermann H., Schmidt S., Janssen J., Koppe C., Knolle P., Castoldi M. (2011). Micro-RNA profiling reveals a role for miR-29 in human and murine liver fibrosis. Hepatology.

[25] Budczies J., Klauschen F., Sinn B.V., Gyorffy B., Schmitt W.D., Darb-Esfahani S., Denkert C. (2012). Cutoff Finder: A comprehensive and straightforward Web application enabling rapid biomarker cutoff optimization. PLoS ONE.

[26] Keane M.G., Shah A., Pereira S.P., Joshi D. (2017). Novel biomarkers and endoscopic techniques for diagnosing pancreaticobiliary malignancy. F1000Research.

[27] Roderburg C., Koch A., Tacke F., Nieuwenhuijsen L., Bruensing J., Vargas Cardenas D., Kreggenwinkel K., Vucur M., Koppe C., Jungebluth P. (2013). Serum concentrations of A Proliferation-Inducing Ligand (APRIL) are elevated in sepsis and predict mortality in critically ill patients. J. Crit. Care.

[28] Wajant H. (2013). The TWEAK-Fn14 system as a potential drug target. Br. J. Pharmacol..

[29] Forero-Torres A., Infante J.R., Waterhouse D., Wong L., Vickers S., Arrowsmith E., He A.R., Hart L., Trent D., Wade J. (2013). Phase 2, multicenter, open-label study of tigatuzumab (CS-1008), a humanized monoclonal antibody targeting death receptor 5, in combination with gemcitabine in chemotherapy-naive patients with unresectable or metastatic pancreatic cancer. Cancer Med..

[30] Pesic M., Greten F.R. (2016). Inflammation and cancer: Tissue regeneration gone awry. Curr. Opin. Cell Biol..

[31] Prabhu L., Mundade R., Korc M., Loehrer P.J., Lu T. (2014). Critical role of NF-κB in pancreatic cancer. Oncotarget.

[32] Liou G.Y., Doppler H., Necela B., Krishna M., Crawford H.C., Raimondo M., Storz P. (2013). Macrophage-secreted cytokines drive pancreatic acinar-to-ductal metaplasia through NF-κB and MMPs. J. Cell Biol..

