# Supplementary figures and images for: Differential Roles of Tumor Necrosis Factor Ligand Superfamily Members as Biomarkers in Pancreatic Cancer
Source: J Clin Med. 2018 Jul 13;7(7):175. doi: 10.3390/jcm7070175 (PMC6068811; doi:10.3390/jcm7070175)

A

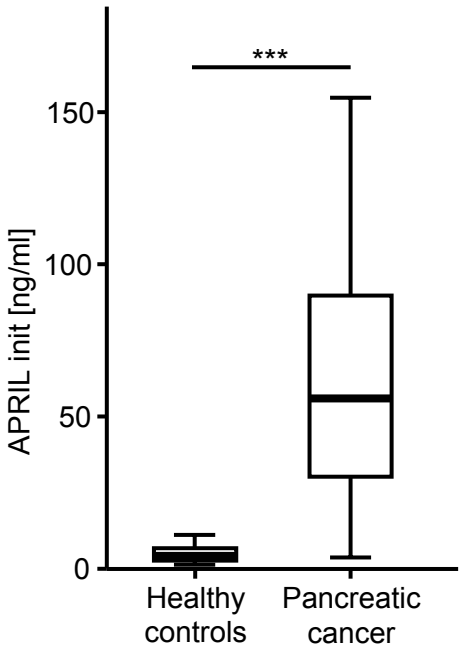

B

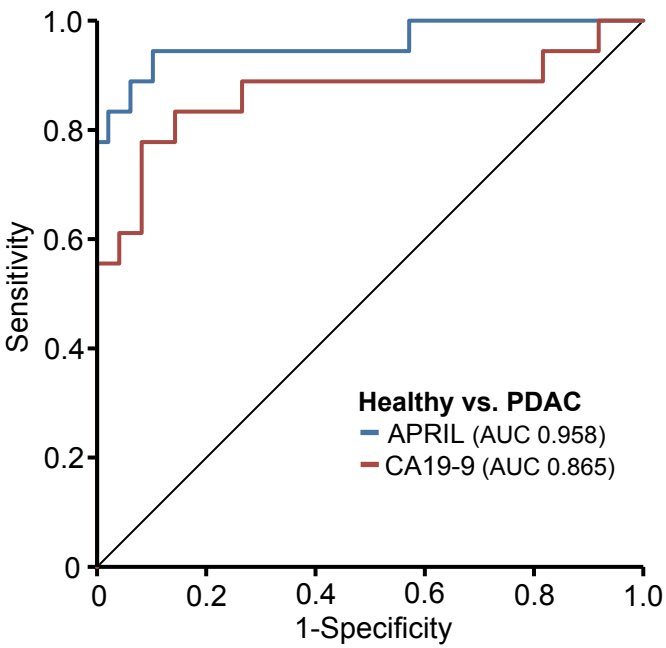

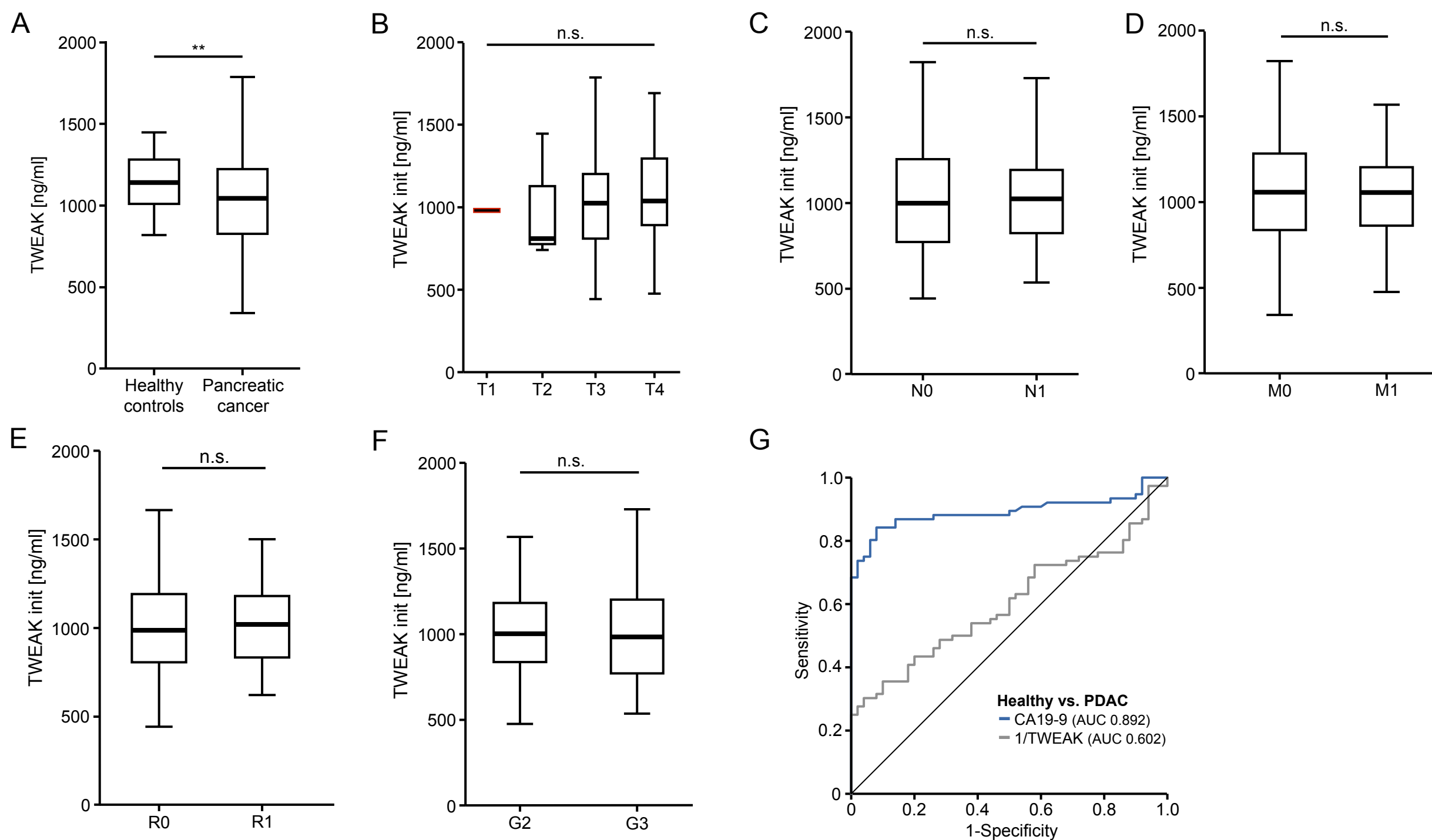

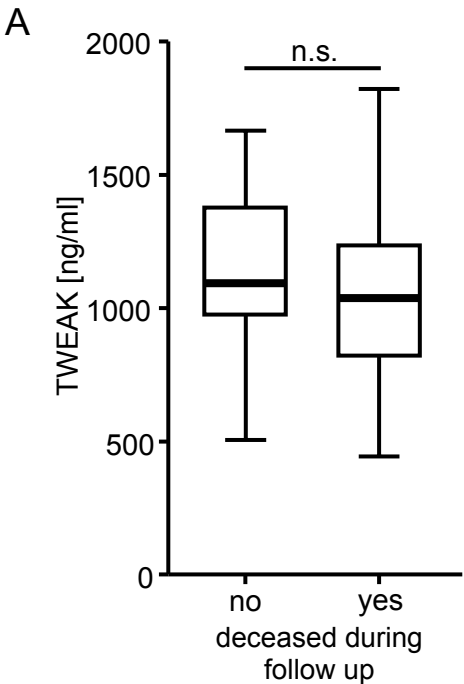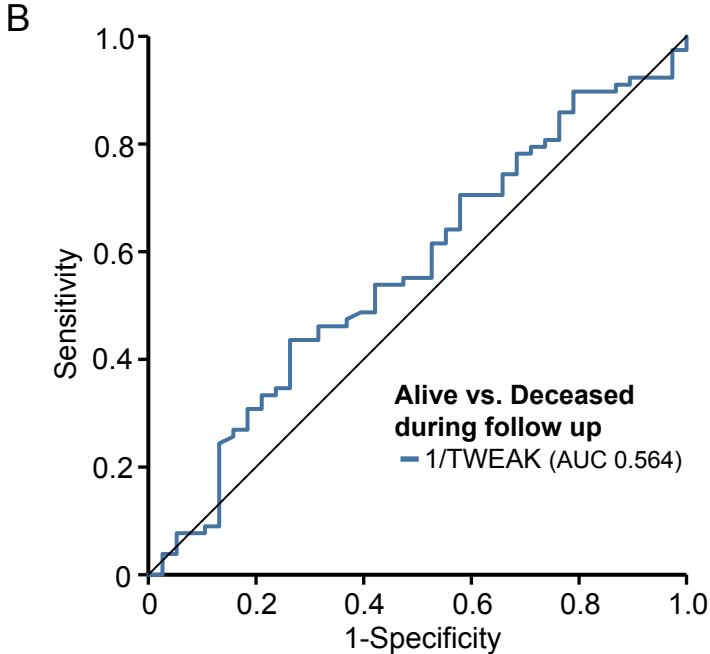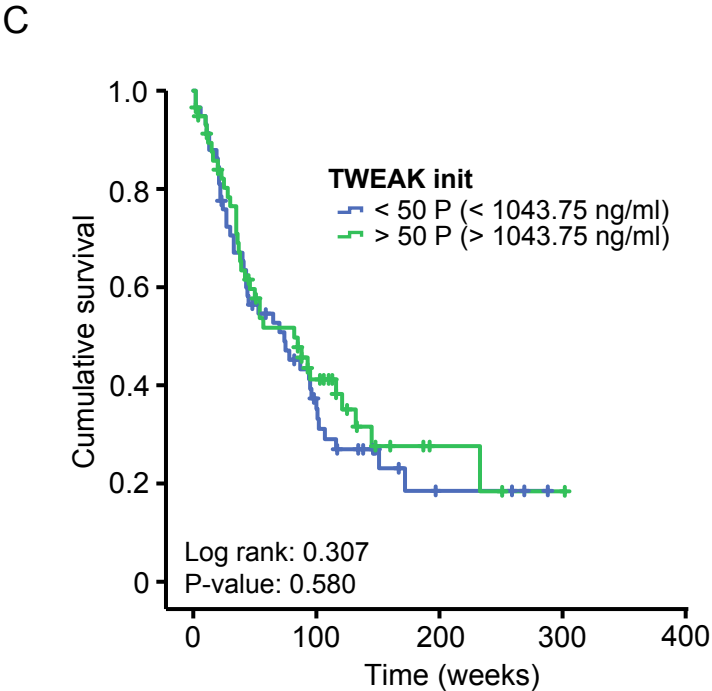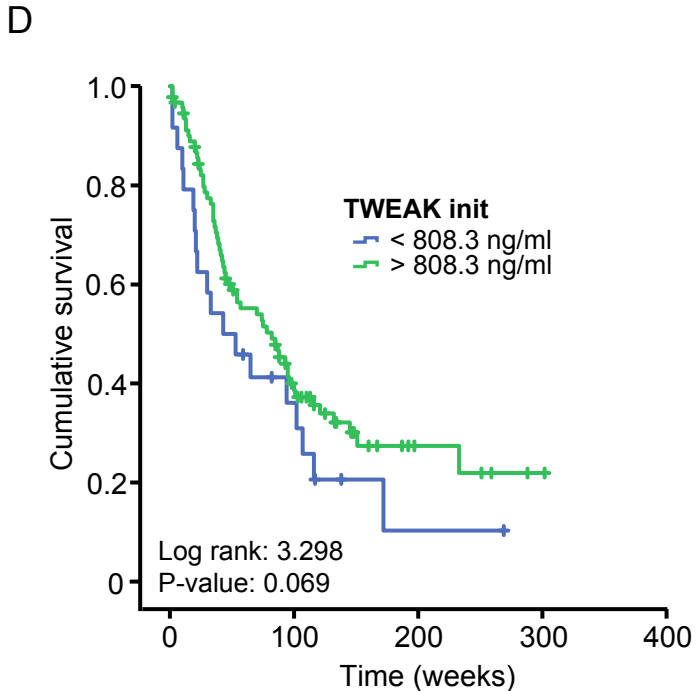

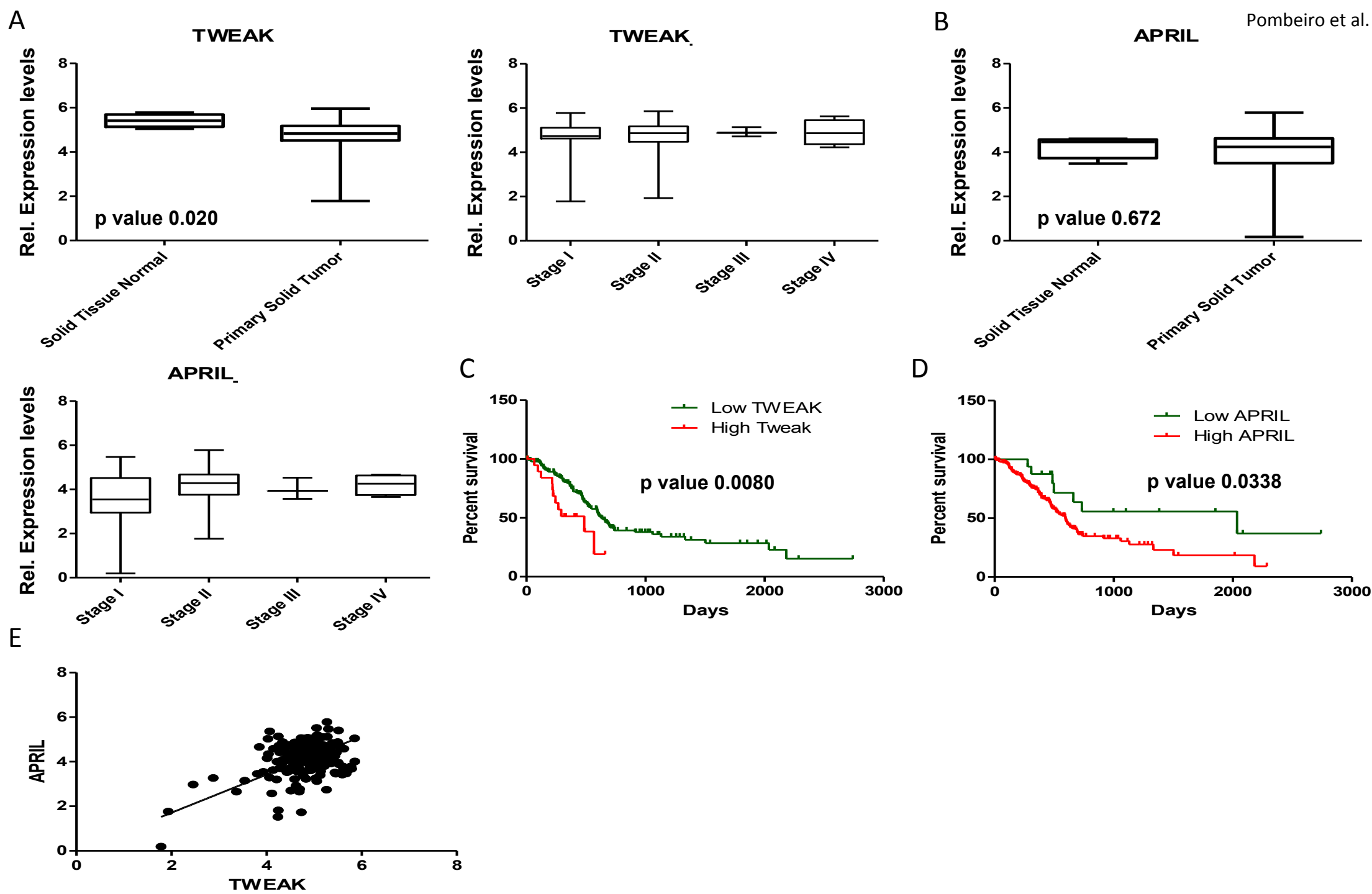

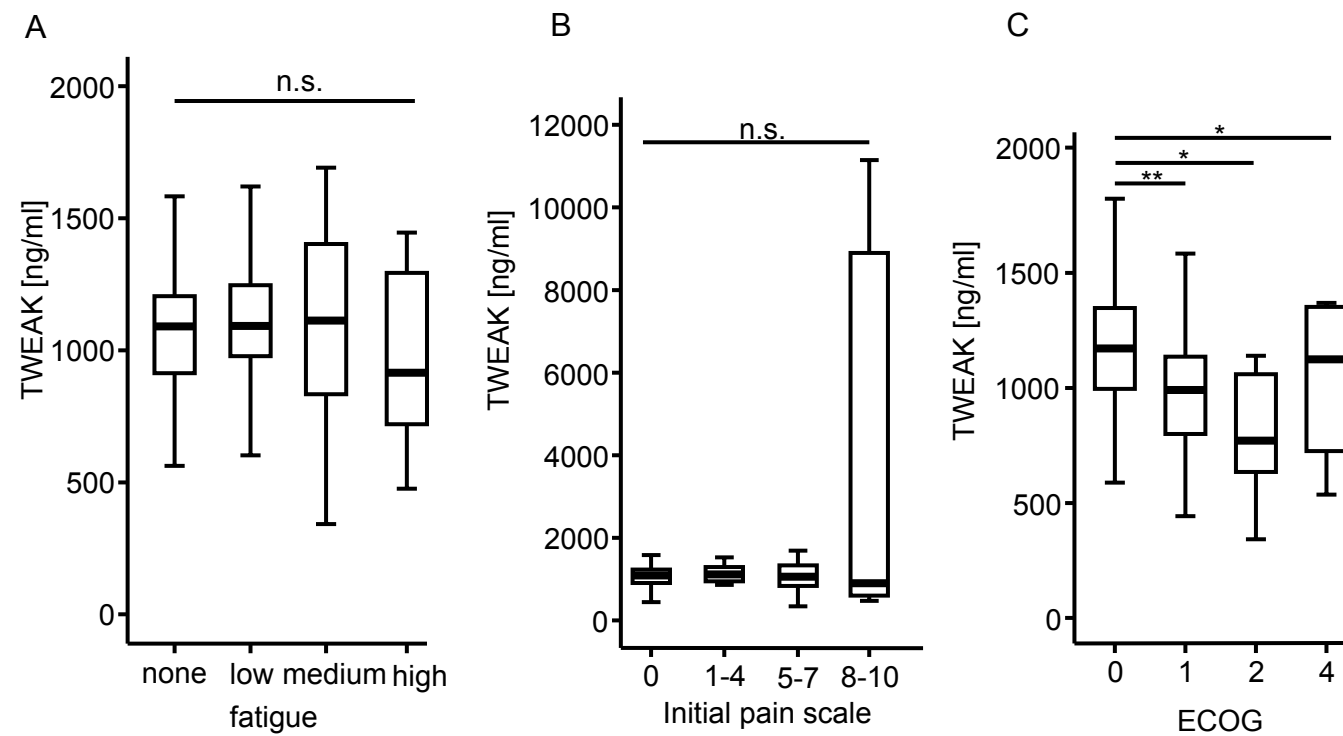

Supplement: Supplementary file 1 [file jcm-07-00175-s001.pdf]
